# Neuronal transcriptional changes associated with incipient Alzheimer's disease neuropathology in entorhinal cortex

**DOI:** 10.1002/alz70855_102023

**Published:** 2025-12-23

**Authors:** Patricia Rodriguez Rodriguez, Wei Wang, Christina Tsagkogianni, Irena Feng, Ana Morello‐Megias, Kaahini Jain, Noemi Vidal, Ana Milosevic, Jean‐Pierre Roussarie

**Affiliations:** ^1^ Division of Neurogeriatrics, Center for Alzheimer Research, Department of Neurobiology, Care Sciences and Society (NVS), Karolinska Institutet, Stockholm, Sweden; ^2^ Rockefeller University, New York, NY, USA; ^3^ Karolinska Institute, Stockholm, Sweden; ^4^ Boston University Chobanian & Avedisian School of Medicine, Boston, MA, USA; ^5^ Hospital de Bellvitge, Barcelona, Spain

## Abstract

**Background:**

Stellate cells from layer II of entorhinal cortex (ECII) are one of the primary sites of pathological tau accumulation and neurodegeneration during preclinical stages of Alzheimer's disease (AD). Little is known about the pathological cascade taking place within these neurons or their neighbors, leading to neurofibrillary tangle formation. Exploring early transcriptional alterations in entorhinal cortex (EC) neurons is essential to find intervention points to curb the disease before symptom onset.

**Methods:**

Here we perform cell‐type specific profiling of human EC at the onset of AD neuropathology, i.e. in postmortem tissue from individuals that were asymptomatic at death. We use single‐nucleus RNAseq as well as fluorescence‐activated neuron nucleus sorting, to determine the molecular changes that accompany pathological tau accumulation. We then use immunofluorescence and multiplex in situ hybridization, to follow up on specific pathways and neuron types.

**Results:**

We identify an expected early response to amyloid pathology by glial cells, in particular by disease‐associated microglia. Importantly, we then provide the first insight into neuronal alterations that coincide with incipient tau pathology: we show evidence that the signaling pathway for Reelin, a putative AD resilience gene, is dysregulated in ECII neurons, while the secreted synaptic organizer molecules NPTX2 and CBLN4, emerging AD biomarkers, are downregulated in surrounding neurons. Investigation of the expression pattern of these genes in control EC, suggests a multicellular mechanism involving different types of neurons from layer II/III.

**Conclusion:**

We highlight here the complex multicellular landscape of EC during the silent phase of AD, when the disease is still largely confined to this region. While the precise mechanisms of pathway dysregulation and the communication between the different highlighted cell types, remain to be discovered, our study paves the way for detailed characterization of the mechanisms governing NFT formation and opens long‐needed novel therapeutic avenues.

